# Synthetic CT in Carbon Ion Radiotherapy of the Abdominal Site

**DOI:** 10.3390/bioengineering10020250

**Published:** 2023-02-14

**Authors:** Giovanni Parrella, Alessandro Vai, Anestis Nakas, Noemi Garau, Giorgia Meschini, Francesca Camagni, Silvia Molinelli, Amelia Barcellini, Andrea Pella, Mario Ciocca, Viviana Vitolo, Ester Orlandi, Chiara Paganelli, Guido Baroni

**Affiliations:** 1 Department of Electronics, Information and Bioengineering, Politecnico di Milano, Piazza Leonardo da Vinci 32, 20133 Milan, Italy; 2 Medical Physics Unit, National Center of Oncological Hadrontherapy (CNAO), Strada Campeggi, 53, 27100 Pavia, Italy; 3 Radiotherapy Unit, National Center of Oncological Hadrontherapy (CNAO), Strada Campeggi, 53, 27100 Pavia, Italy; 4 Department of Internal Medicine and Medical Therapy, University of Pavia, 27100 Pavia, Italy; 5 Bioengineering Unit, National Center of Oncological Hadrontherapy (CNAO), Strada Campeggi, 53, 27100 Pavia, Italy; 6 Clinical Unit, National Center of Oncological Hadrontherapy (CNAO), Strada Campeggi, 53, 27100 Pavia, Italy

**Keywords:** synthetic CT, MRI guidance, MRI-only, image-guided radiotherapy, carbon ion radiotherapy, particle therapy, deep learning

## Abstract

The generation of synthetic CT for carbon ion radiotherapy (CIRT) applications is challenging, since high accuracy is required in treatment planning and delivery, especially in an anatomical site as complex as the abdomen. Thirty-nine abdominal MRI-CT volume pairs were collected and a three-channel cGAN (accounting for air, bones, soft tissues) was used to generate sCTs. The network was tested on five held-out MRI volumes for two scenarios: (i) a CT-based segmentation of the MRI channels, to assess the quality of sCTs and (ii) an MRI manual segmentation, to simulate an MRI-only treatment scenario. The sCTs were evaluated by means of similarity metrics (e.g., mean absolute error, MAE) and geometrical criteria (e.g., dice coefficient). Recalculated CIRT plans were evaluated through dose volume histogram, gamma analysis and range shift analysis. The CT-based test set presented optimal MAE on bones (86.03 ± 10.76 HU), soft tissues (55.39 ± 3.41 HU) and air (54.42 ± 11.48 HU). Higher values were obtained from the MRI-only test set (MAE_BONE_ = 154.87 ± 22.90 HU). The global gamma pass rate reached 94.88 ± 4.9% with 3%/3 mm, while the range shift reached a median (IQR) of 0.98 (3.64) mm. The three-channel cGAN can generate acceptable abdominal sCTs and allow for CIRT dose recalculations comparable to the clinical plans.

## 1. Introduction

In the treatment of abdominal tumors such as those of the liver and pancreas, carbon ion radiotherapy (CIRT) is considered a promising therapeutic option, thanks to its excellent geometrical selectivity and radiobiological effectiveness [1,2]. However, tumors subject to respiratory motion may suffer from inter- and intra-fraction motion that needs to be properly accounted for during planning and delivery. So far, the repeated acquisition of computed tomography (CT), in the form of respiratory-correlated 4DCT, is the clinical routine for motion management, but concurrently exposes the patient to additional non-therapeutic radiation [3].

The growing interest in magnetic resonance imaging (MRI) in recent years has fostered the development of MRI-only workflows that would guarantee the absence of ionizing radiation while exploiting the superior soft tissue contrasts of MRI scans. In this regard, online MR-guided radiotherapy (MRgRT) is a clinical reality in conventional radiotherapy [4,5,6], while no integrated system is clinically available for online MR guidance in particle therapy (PT), with only a few feasibility studies addressing protons but not carbon ions [7,8]. Nonetheless, the off-line use of MRI in CIRT may support treatment planning and adaptation through the generation of synthetic CT (sCT), used as alternatives to verification CTs, and avoid non-therapeutic radiation along with CT-MRI registration errors that are typically relevant in the abdominal site [9,10]. Different methods (i.e., bulk density override, atlas based, voxel based) have been studied to generate sCTs starting from MRI scans [8,11]. As an alternative, deep learning (DL) methods have been largely investigated [11,12,13,14,15,16,17], relying on their capability of autonomously learning hidden relationships among data [18]. In particular, deep convolutional neural networks (DCNN) showed promising results for the generation of sCTs of the head and neck with respect to conventional methods, as they are able to catch complex nonlinear relationships among MRI and CT [12,15]. Generative adversarial networks (GAN), especially in the conditional form (conditional GAN—cGAN) have been instead considered for more complex anatomical districts such as the abdomen, thanks to the adversarial learning process, which provides great efficacy in image-to-image translation applications [19,20,21]. The main limitation of these networks is the need for paired MRI-CT samples to perform the training, which is a critical aspect when dealing with multi-modal volumes. In recent years, new architectures have been proposed to overcome the need for paired CT-MRI training datasets, with cycleGAN and fully bidirectional networks showing great results in end-to-end synthetic image generation tasks [22]. Clearly, the use of such complex and multi-network architectures requires high dimensional training datasets, which still remain an evident concern in most clinical realities. The use of multiple MRI sequences (e.g., Dixon in-phase (IP), opposed-phase (OP), fat, and water) has been also investigated to increase the quality of critical anatomical structures, but no relevant improvements were shown [23].

The dosimetric evaluation of DL-based sCTs has been investigated for X-ray and proton radiotherapy in most anatomical districts [20,21,22,23,24,25,26,27,28], while a similar analysis for CIRT applications is still missing in the literature.

In the treatment of abdominal targets, the presence of inter- and intra-acquisition motions, which mainly translate into the different dispositions of the air fillings in the organs, highly affects the dose distribution for carbon ions plans [29]. An MRI-only workflow for CIRT would thus require more stringent constraints and tolerances in terms of accuracy in the definition of HU values, if compared to conventional radiotherapy, since a precise knowledge of particle stopping power, estimated from HU values within the patient anatomy, is essential for accurate treatment planning, to limit any range shift and damage to healthy tissues [30,31]. Up to now, to the authors’ best knowledge, the application of sCT to CIRT plans has been investigated only by Knäusl et al. [32] for head and neck imaging, while no study has been performed on CIRT plans for abdominal tumors.

In this study, we focused on the generation of abdominal sCTs through a cGAN, with the final aim of simulating an MRI-only workflow for CIRT, based on recalculation of the clinical CIRT treatment plans for generated abdominal sCT volumes. This study, therefore, was conducted to evaluate, for the first time, the feasibility of sCT in CIRT for abdominal sites.

## 2. Materials and Methods

### 2.1. Patient Cohort

Image datasets were collected from 24 patients affected by liver or pancreatic cancer and treated with CIRT at the National Centre for Oncological Hadrontherapy (CNAO, Pavia, Italy) between 2017 and 2021. Standard clinical workflow comprised the acquisition of a 4DCT followed by a 3D MRI on the same day, for contouring and planning preparation. The same immobilization setup, consisting of customized pillows (MOLDCARE Cushion, QFix, Avondale, PA, USA) and non-perforated body thermoplastic masks (Klarity Medical Products, Heath, OH, USA), was used both for CT and MRI acquisitions and treatment delivery. Acquisition in two different scanners along with re-positioning of the patient with the thermoplastic mask caused inter-acquisition motion. For 15 patients, re-evaluative images (both CT and MRI) were acquired during the treatment course with the same immobilizations setup and were considered independent from the first acquisition, leading to a total of 39 CT-MRI volume pairs collected. The study was approved by the local ethical committee, and all patients signed the informed consent (CNAO 37-2019 4D-MRI).

The 4DCTs were acquired during patient free breathing on a Siemens SOMATOM Sensation Open CT scanner (resolution 0.98 × 0.98 × 2 mm^3^). Clinical plans were optimized at end-exhale for gated treatments; as such, only this phase was used in this work to derive sCTs. CT acquisitions had a variable number of slices, resulting in a volume size of 512 × 512 × [96 − 145] voxels. MRI acquisitions were performed with a Siemens Magnetom Verio 3T scanner. Three-dimensional breath-hold T1-weighted volumetric interpolated breath-hold examination (VIBE) sequences were acquired at end-exhale with 1.06 × 1.06 × 3 mm^3^ resolution (repetition time TR = 3.87 ms, echo time TE = 1.92 ms). For two patients, MRI acquisitions had a voxel size of 1.25 × 1.25 × 3 mm^3^ and 1.125 × 1.125 × 3 mm^3^. Most of the MRI acquisitions had 320 × 260 × 64 voxels, except for one having 88 transversal slices. Two CT-MRI volume pairs were discarded from the study because of the low quality of the acquired images. Therefore, 37 volume pairs were used: 32 pairs were exploited for cross-validation (CV) and training, while five pairs were randomly selected and held out for testing (Table 1) for more details on the treatment plans. All treatment plans were optimized with the RayStation (Raysearch Laboratories, Stockholm, Sweden—version 10.B) Treatment Planning System (TPS) on the end-exhale reconstructed CT phase and clinically approved. Corresponding organs at risk (OARs), gross tumor volume (GTV) and clinical target volume (CTV) were segmented by radiation oncologists. The relevant OARs included were kidneys, aorta, colon, duodenum, stomach and spinal cord.

### 2.2. Pre-Processing

Although CT and MRI scans were acquired the same day and with an immobilization setup, inter-acquisition motion (i.e., anatomical changes between CT and MRI scans) was present. A manual rigid registration was firstly performed to align CT and MRI scans. The application of deformable image registration (DIR) was investigated but did not show relevant improvements on the quality of sCTs; therefore, it was not applied (Supplementary Material S1). Indeed, in most cases, the multi-modal DIR could hardly compensate for air filling mismatches and caused large bone deformations and artefacts that reduced the size of the training dataset, making it a less effective approach. Nonetheless, given the lack of a real ground truth (i.e., a CT with MRI anatomical configuration), DIR was applied for the generation of pseudo ground truths (see Section 2.4).

CT slices were resampled to the corresponding MRI spacing to guarantee the voxel consistency among the two volumes and clipped to [−1000,+1047] HU as previously performed by Maspero et al. [14] to reduce the discretization step, while background values were set to −1000 HU. For MRI volumes, the pre-processing consisted of: (i) bias field correction to reduce low frequency noise due to magnetic field inhomogeneities [33]; (ii) reduction of Gaussian noise through a non-linear bilateral filter; (iii) contrast enhancement through histogram clipping to 99th percentile of intensity [14,21,24]: (iv) setting of background values to zero; and (v) histogram matching to similarly distribute grey levels across all MRI volumes [34].

CT and MRI preprocessed volumes were all resized to 256 × 256 to match the network input dimensions [19]. Then, the input MRI and target CT transversal slices were segmented in three channels by means of CT-based masks (i.e., air [−1000,−800] HU, bone [150,1047] HU, soft tissues [−800,150] HU) and then linearly scaled to [−1,1].

As a final step, eight transformations were applied to each CT/MRI channel triplet used for training, including horizontal or vertical flip, Gaussian noise adding, shear, rotation and cropping. Thus, the initial training dataset composed of 2014 CT-MRI slice pairs was enlarged to 16,112. Pre-processing was performed through Python scripts implemented using SimpleITK modules (version 2.0.2).

### 2.3. Neural Network

The neural network used in this work consisted of a cGAN derived from the open-source network “Pix2Pix” by Isola et al. [19], adapted to work on three channels (air, bone, soft tissues) to better account for the anatomical complexity of the abdomen [35] and to deal with the limited dataset of 39 volume pairs.

**Figure 1:**
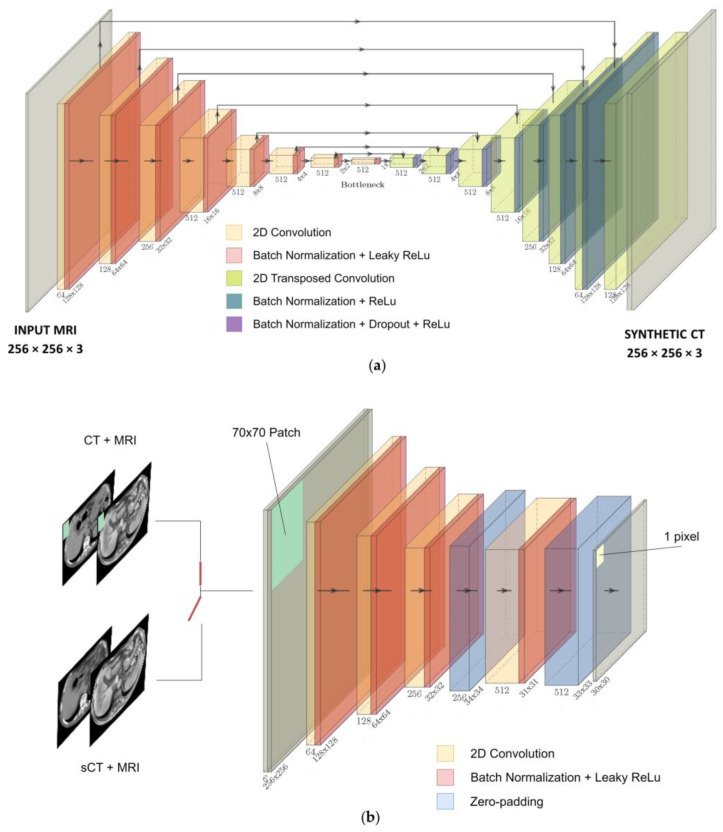
(**a**) U-net generator. (**b**) PatchGAN discriminator.

The net was trained on transversal slices and composed of a 256 × 256 × 3 U-net generator (Figure 1a), used to generate sCTs, and a 70 × 70 × 3 PatchGAN discriminator (Figure 1b), used to judge the quality of the output with high resolution during the training [19]. The U-net is composed of eight encoder blocks, each comprising convolution, batch normalization and leaky rectified linear unit (ReLU) layers, and seven decoder blocks, each composed of transposed convolution, batch normalization, dropout and ReLU layers. The PatchGAN architecture is made of four encoding blocks, such that each pixel of the 30 × 30 output classifies a 70 × 70 pixel patch of the input image. For the detailed architecture, refer to Figure 1a,b.

The loss function (Equation (3)) combined *L_1_* norm (Equation (1)) and cGAN adversarial cross-entropy loss (Equation (2)) to reduce blurring effects and artefacts [19]:

(1) $$L_{1}=E_{x,y,z}\left[{\left\|x-G\left(y,z\right)\right\|}_{1}\right]$$

(2) $$L_{cGAN}=-\left[E_{y,x}\left[\log D\left(y,x\right)\right]+E_{y,z}\left[\log\left(1-D\left(y,G\left(y,z\right)\right)\right)\right]\right]$$

(3) $$L_{tot}=L_{cGAN}\left(\theta_{g},\theta_{d}\right)+\;\lambda \cdot L_{1}(\theta_{g})$$

where *x* is the target CT, *y* is the input MRI, *z* the noise, 𝐺(𝑦,𝑧) the sCT, 𝐷(𝑦,𝑥) the PatchGAN output, 𝜃𝑔, 𝜃𝑑 are the trainable parameters of generator and discriminator, and λ = 100 is a weight for the *L_1_* norm, as from Isola et al. [19]. The role of this additional loss is to have the generator not only mislead the discriminator, but also generate synthetic images that mimic the target CT in an *L_1_* sense, reducing the blurring and improving the representation of structures. The cGAN loss was evaluated on the channel triplets, while the *L_1_* norm was evaluated after reassembling the sCT slice.

The MRI represents the conditional input of the net, with the noise z being provided in the form of dropout on several layers of the U-net generator during training and testing. The training was performed by alternating one gradient descent step on the discriminator and one on the generator, using ADAM optimizer with momentum parameters β1 = 0.5, β2 = 0.999. The training was stopped after 20 epochs, which were sufficient to achieve convergence thanks to data augmentation.

The cGAN was optimized through a six-fold cross-validation (CV), setting the batch size to 1 and discriminator learning rate to 2 × 10^−7^ (Supplementary Material S2). This confirmed that the instance normalization (i.e., the use of batch-normalization layers with batch = 1) is well-suited for image generation tasks [36]. The network was trained on the full dataset (i.e., 32 volumes) and tested on the remaining five volumes (Figure 1c). Before stacking all of the output slices to rebuild the synthetic volumes, the values of each channel were re-scaled to the corresponding HU range and re-assembled by means of the same masks used for the channels’ segmentation. The stacked volumes were finally resized to the original MRI [320 × 260 × N_slices_] size.

### 2.4. Experiments

The results were evaluated by means of similarity metrics such as mean absolute error (MAE), root mean squared error (RMSE), normalized cross correlation (NCC), structural similarity index (SSIM) and peak signal-to-noise ratio (PSNR) between sCT and target CT (Supplementary Material S3), with the exclusion of the background, both within CV and testing scenarios. All metrics were evaluated on the basis of reassembled volumes, applying the corresponding tissue mask. Specifically, the five held-out patients were used (i) to create the test set, where CT-based masks were used for the analysis, and (ii) to build an MRI-only simulation set, where the use of CT-based masks was replaced by manual segmentation of the three channels directly on MRI (Figure 1c).

**Figure 2:**
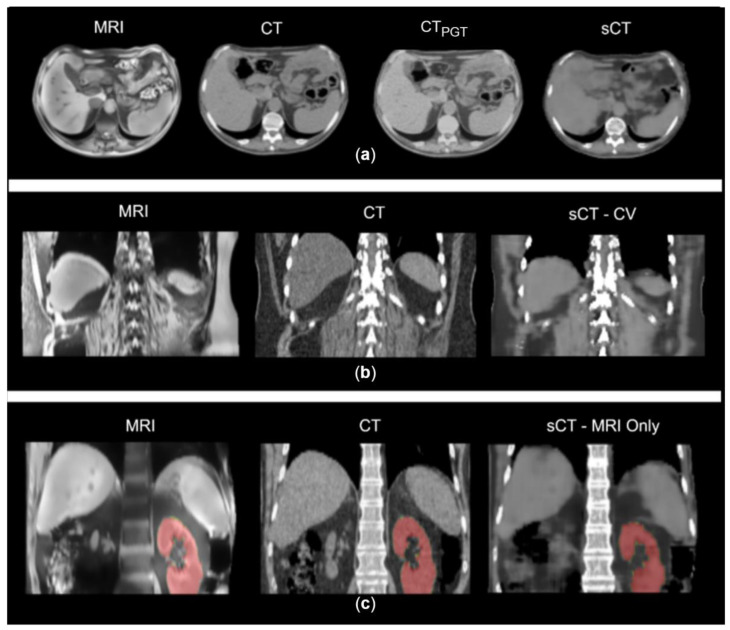
(**a**) Example of MRI, CT, sCT and pseudo ground truth (CT_PGT_) on axial plane. CT_PGT_ still shows visible discrepancies with MRI anatomical condition. (**b**) Example of inter-acquisition motion in a CT-MRI pair and the resulted sCT in CV. (**c**) Example of MRI, planning CT and synthetic CT from MRI-only scenario. In red, the segmentation of kidneys used for the geometrical analysis.

Within the MRI-only workflow, the evaluation of similarity metrics was performed between sCTs and reference volumes obtained by applying DIR between the target CT and MRI (i.e., pseudo ground truths, CT_PGT_) to compensate for MRI-CT inter-acquisition motion (Figure 2a).

The MRI-only scenario was evaluated also through geometrical criteria: dice coefficient (DSC), 95th percentile Hausdorff distance (HD) and the center of mass distance (CoMD) were calculated on kidney segmentations of MRI, CT and sCT, to assess the quality of the sCT in terms of correct reproduction of soft tissues with respect to the MRI anatomy. HD, DSC and CoMD were assessed for each couple of segmented volumes (CT-MRI, sCT-CT, sCT-MRI). sCTs were compared to MRIs since we expected the sCT to be representative of the MRI anatomical condition; CT was compared to MRI to determine the initial mismatch, while sCTs were compared to target CTs to confirm that sCT was far from matching CT structures.

Due to the lack of a real ground truth, the net was also validated on a CT-MRI volume pairs obtained through a computational phantom (i.e., XCAT [37] for CT and correspondent ComBAT [38] for MRI) that guaranteed an improved match of anatomical structures between the two volumes, avoiding any inter-acquisition motion (Supplementary Material S4).

The clinical CIRT plans were recalculated on the MRI-only sCT for each patient through the TPS and evaluated on the basis of DVH-based metrics (GTV D_95%_, CTV D_95%_, D_2%_ on OARs) and dose difference maps with respect to the original plan. A two one-sided test of equivalence for paired samples (TOST-P) was used to compare the DVH metrics, considering a confidence interval of 95% and an equivalence interval of ±0.5%. The global gamma analysis was also performed with 1 mm/1%, 2 mm/2%, 3 mm/3% as tolerance criteria, and three dose thresholds: 10%, 50%, and 90% on the prescription dose. A range shift (*RS*) analysis was performed to take into account possible dose shifts, which are generally averaged in gamma analysis [15,39]. In this regard, both range shift (*RS*) and relative range shift (*RRS*) were evaluated on a beam-by-beam basis, considering an acceptability threshold set to 5 mm, in accordance with clinical margins used at CNAO [40]. RS and RRS are defined as:

(4) $$RS=(R_{sCT80}-R_{CT80})$$

(5) RRS=(RSRCT80)

where *R_sCT80_* and *R_CT80_* are the beam ranges computed at 80% of the dose peak. This evaluation was performed on the dose profile along the central axis of each beam, considering 10 transversal slices, for a total of 60 RSs.

All computational steps were performed on a Precision 5820 Tower DELL workstation equipped with a 16 GB RAM Nvidia GPU (QUADRO P5000). A full training procedure took around 12 h, while the generation of a synthetic volume took ~5 s.

## 3. Results

The similarity metrics from CV, testing and MRI-only simulation are presented in Table 2. Comparable results were found between CV and test performance, with MAE on soft tissues and air channels being lower than that on bone. Notwithstanding inter-acquisition discrepancies (Figure 2b), the air channel showed an error of 54.42 ± 11.48 HU, the soft tissues 55.39 ± 3.41 HU, and the bone structures 86.03 ± 10.76 HU.

The results relative to the MRI-only evaluation were characterized by larger values on all metrics. In particular, the errors grew to 279.01 ± 142.46 HU on the air channel, 154.87 ± 22.90 HU in the case of bone structures, and 88.22 ± 9.88 HU within the body.

The geometrical analysis of the MRI-only workflow (Figure 2c) showed the lowest discrepancies between CT and MRI segmentation, confirming good accuracy in replicating MRI anatomy. As an example, the average CoMD in sCT-MRI was 5.85 ± 4.87 mm versus 7.75 ± 5.92 mm in CT-MRI and 13.30 ± 10.42 mm for sCT-CT. Detailed results are reported in Supplementary Material S5.

The validation performed on the phantom showed an MAE of 73.3 HU on the whole volume, while the MAEs on the three channels were of 66.11 HU, 77.93 HU and 167.36 HU for soft tissues, air, and bones, respectively (see Supplementary Material S4).

**Figure 3:**
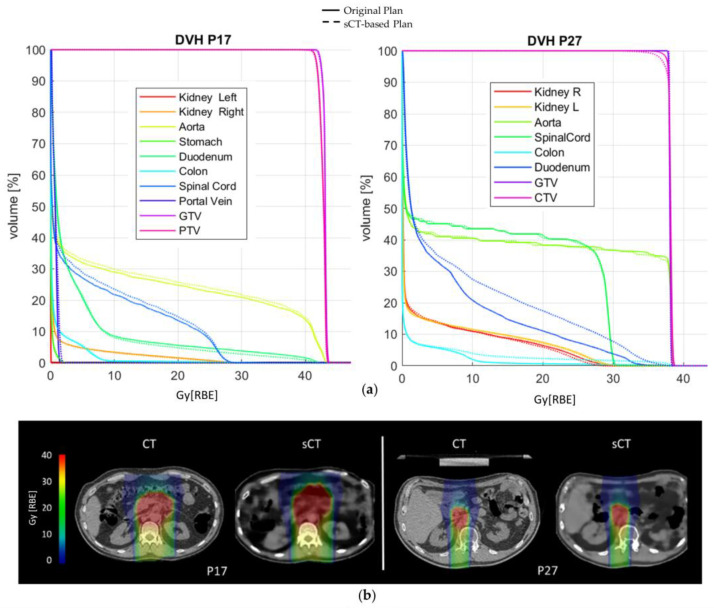
(**a**) DVH comparison on patients P17 and P27; (**b**) original CIRT plan (RBE) and sCT-based recalculation for patients P17 and P27.

**Figure 4:**
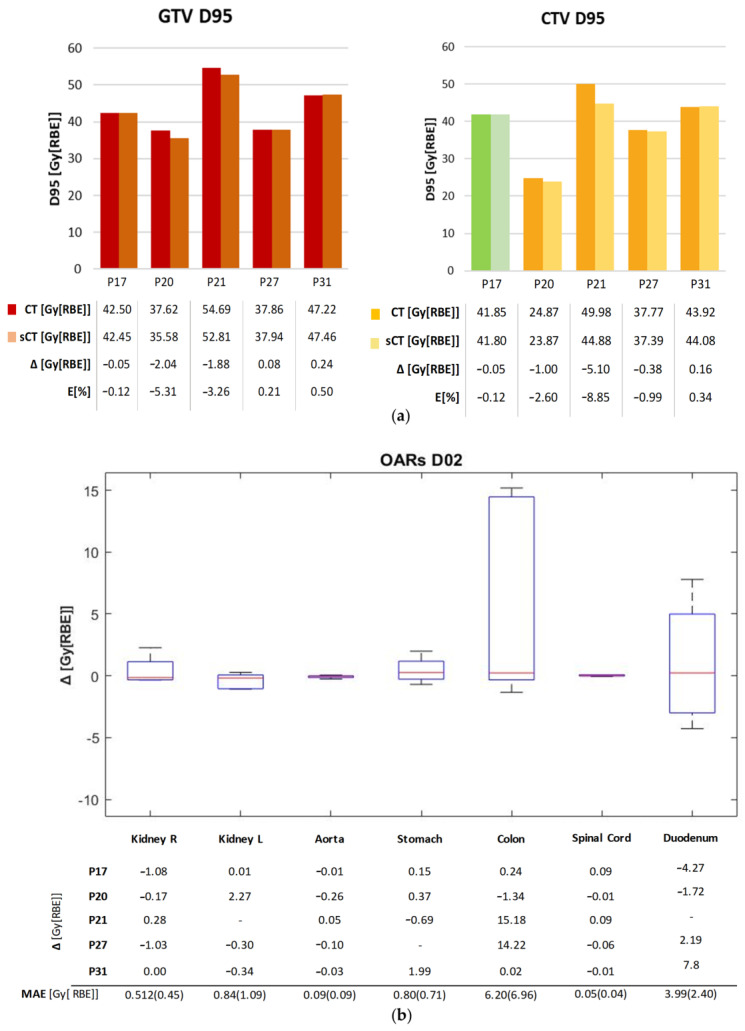
(**a**) D95% values for GTV and CTV in the original plan (CT) and the recalculated one (sCT). For P17, PTV was considered in this comparison (shown in Green). The table contains the dose values and the dose difference Δ[Gy[RBE]], as well as the error relative to the prescribed dose E [%]. (**b**) D2% difference (sCT-CT) for the main OARs on each patient, and the D2% MAE over each OAR. The red mark indicates the median, and edges of the box show the 25th and 75th percentiles.

As for dose accuracy, Figure 3 shows the DVH comparison for patient and dose recalculations on P17 and P27 (complete results reported in Supplementary Materials S6–S8). The GTV and CTV D_95%_ as well as the D_2%_ on the organs at risk (OARs) are displayed in Figure 4, expressed in terms of dose difference Δ(sCT-CT), MAE and relative error (E[%]) with respect to the prescribed dose. For all five patients, good reproducibility was shown relative to the dose to the GTV, with patients P20 and P21 being characterized by errors of −2.04 Gy[RBE] and −1.88 Gy[RBE] respectively, corresponding to −5.3% and −3.3% of the prescribed dose. The MAE on the GTV D_95%_ was 0.86 ± 0.90 Gy[RBE].

Similarly, the values relative to the CTV showed the two dose distributions to have comparable results, with a maximum error of −5.10 Gy[RBE] for P21 (−8.9% of the prescribed dose). The MAE on the D_95%_ CTV was 1.34 ± 1.33 Gy[RBE]. The D_2%_ relative errors on OARs lay in an interquartile range (IQR) of [−0.24,0.22]%, although the colon reached a peak relative error of 37.03% (14.22 Gy[RBE]), and the duodenum of 16.25% (7.8 Gy[RBE]) (Supplementary Material S6). The voxel-wise dose difference maps are shown in Figure S5 in Supplementary Material S7, highlighting the worst-case slice for each test patient. Regions with a high dose difference can be mainly seen with correspondence of air pocket mismatches, while the overall distribution of dose to the body is comparable. Indeed, as in Table S6 in Supplementary Material S7, all test patients showed a median dose difference close to zero, with the widest IQR being 0.158 Gy[RBE] on patient P21. The maximum errors were in the range [22.64,42.36] Gy[RBE]. In this regard, an incomplete reproduction of the kidney affected the dose distribution in patient P27 (Figure S6), while the limited field of view of MRI introduced dose artefacts on recalculation for patient P21 (Figure S7).

A peak gamma pass rate of 94.88% was obtained in the 3%/3 mm analysis (Supplementary Material S9).

**Figure 5:**
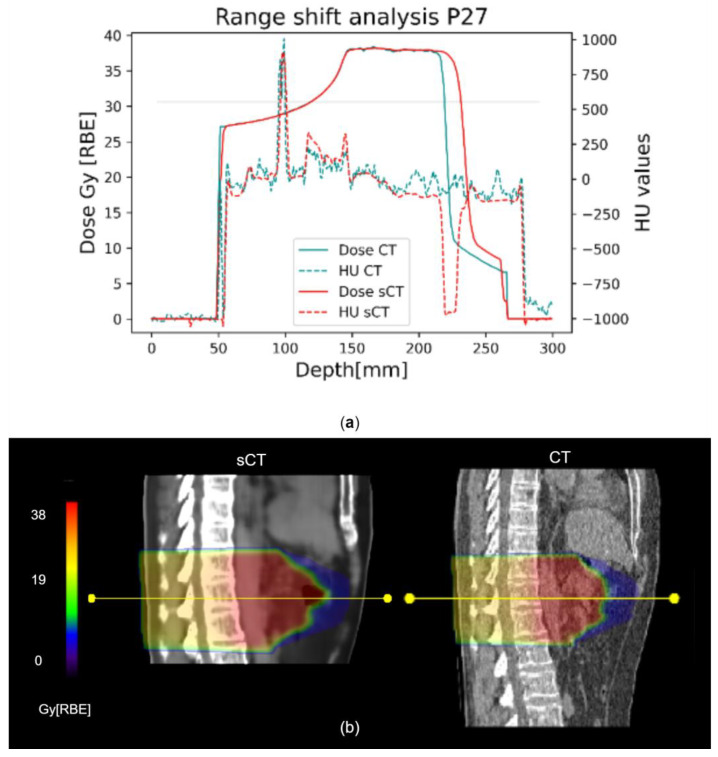
Representative range shift analysis on patient P27. (**a**) The graph shows both dose and HU profiles in CT (light blue) and sCT (red), evaluated along the yellow line shown in (**b**). The 80% reference is marked by the horizontal line. (**b**) Corresponding CT and sCT sagittal views are compared. The yellow line is one of the 10 considered for each beam, on different transversal slices.

The range shift analysis showed a median (IQR) RS of 0.98 (3.64) mm and RRS of 0.61 (2.14)% (Figure 5). Considering each beam individually, as shown in Table S9 in Supplementary Material S10, patient P27 was the one showing the highest errors, with a median RS of 5.69 (6.97) mm.

## 4. Discussion

In this work, we investigated for the first time the feasibility of a cGAN in generating sCTs of the abdominal site for applications in CIRT. The cGAN, trained with transversal CT-MRI slice pairs, was optimized to work on three channels corresponding to air, bone and soft tissues to better account for the anatomical complexity of the abdomen.

The performance of the network was firstly evaluated on the basis of similarity metrics of the test set built with CT-based segmentation. Despite MRI to CT inter-acquisition motion, the MAE on the body (57.08 ± 2.79 HU) was comparable to results obtained by other works on the abdominal site that used multiple Dixon sequences and more complex architectures (55.56 ± 2.27 HU [26] and 60.42 ± 2.27 HU [23]), as well as U-nets trained on T1w–T2w MRI acquisitions (62 ± 13 HU) [24]. In addition, this work favorably compares to other studies exploiting cGAN or cycleGAN in terms of MAE_BODY_ [20,21,25], even if the bidirectional network from Xu et al. achieved outstanding results, although working on a much wider unpaired dataset [22]. The NCC (0.92 ± 0.02) was shown to be consistent with those of other works [23,25,26], while the PSNR (27.64 ± 0.68 dB) was comparable to the work by Fu et al. [21]. Optimal MAE metrics were obtained in the generation of bone structures (86.03 ± 10.76 HU) and soft tissues (55.39 ± 3.4 HU), outperforming other works in the literature [20,24,25,28].

The MAE on the air channel and bones was also low with respect to other approaches with cycleGAN [25]. This could be due to the use of CT-derived masks on the test set, which may have aided the replication of CT air pockets and bone structures on the sCTs.

In order to cope with this and to simulate a real-case scenario, the network was then tested on an MRI-only simulation set, where the MRI segmentation was completely independent from the use of CT. Given the limited performance of multi-modal DIR on the considered dataset, manual segmentation of the three channels on MRI was considered the most accurate approach, despite the time-consuming task for clinical purposes. Nonetheless, DIR was applied between the target CT and MRI to overcome the lack of a ground truth CT representative of MRI anatomical condition, notwithstanding the minor contribution of such registration. Indeed, this process did not fully compensate for different air cavities and inter-acquisition motion; therefore, the volumes (i.e., CT_PGT_) used as reference were still showing visible discrepancies with respect to the MRI, biasing the evaluation of the performance of the network (Figure 2a). A similar consideration applied by Florkow et al. highlighted errors in HU intensities caused by inter-acquisition variations that were not compensated by the deformable registration [24]. Moreover, the manual segmentation of MRI represented a complex step, especially for bones, since they are not clearly visible on VIBE volumes. Nonetheless, the results were shown to still be acceptable when compared to the literature, with MAE on soft tissues (75.00 ± 8.12 HU) being aligned to results obtained with GAN (90 ± 29 HU) or cycleGAN (58.62 ± 30.61 HU) [25,27].

The geometrical analysis, performed on the MRI-only test set, was conducted to support the good geometrical agreement between sCT and MRI: in general, sCT and MRI segmentations showed the best match, confirming the expected performance of the net in reproducing the MRI anatomy, while sCTs showed higher deviations with respect to the target CT scans. This evaluation was performed on segmentations of the kidneys, which are well contrasted organs, and can be considered representative of the geometrical accuracy in the reproduction of soft tissues.

Due to the lack of a proper ground truth on patients’ data, we supported our results with a validation performed on a single CT-MRI volume pair obtained through a computational phantom (i.e., XCAT [37] for CT and correspondent ComBAT [38] for MRI). This approach, which guaranteed the perfect anatomical match between the two volumes, provided promising outcomes (i.e., MAE of 73.3 HU on the whole volume) aligned with the presented results and the literature. The MAE on the three channels described a discrete reproduction of the air fillings and soft tissues, with poorer performance on bones, as noticed also in the literature. NCC reached 0.89, describing an acceptable reproduction of the anatomical structures (see Supplementary Material S4).

For dose analysis, most patients (Figure 4a and Supplementary Materials S6–S8) presented DVH metrics within clinical tolerances (i.e., below 3% of the prescribed dose) and aligned with studies in the literature on protons (relative error < 3% on PTV [25] and relative error < 2% on ITV—the internal target volume [24]). Moreover, *p*-values gave statistical evidence of the equivalence between the DVH metrics from sCT and CT (*p* < 0.05, Table S7 in Supplementary Material S8); however, a larger test dataset would make these considerations more solid.

Concerning DVH metrics, Liu Y. et al. for protons [25] as well as Liu L. for photons [28] obtained promising results, with errors on the maximum dose on the PTV being in the range [−1,+1] Gy[RBE], also thanks to the use of lateral beams that avoided most of the air-filled organs. Florkow et al. [24] obtained acceptable errors on the OARs, with D_2%_ being in the range [−2.7,3.7]% of the prescribed dose. Notwithstanding the use of DIR, the work by Florkow et al. suffered from inter-scan variations (i.e., air fillings), that may have caused an overestimation of the actual differences between the planning CT and sCT [24]. In our work, the IQR on the D_2%_ relative error was [−0.24,0.22]%, but high errors were highlighted on the colon (37.03%) and the duodenum (16.25%, Figure 4c), which were highly affected by inter-acquisition motion of air fillings between sCT (representative of MRI anatomy) and planning CT. In this regard, the work by Knäusl et al. [32] showed that the constraints on OARs are very challenging for compliance, presenting errors of up to 28%, mostly due to the incorrect representation of bones or air cavities. Discrepancies in the dose distributions were also confirmed by the gamma analysis, which in our work showed a peak value of 94.88 ± 4.9% against the 99.37 ± 0.99% reported in the literature [25]. Table S8 in Supplementary Material S9 shows a comparison of gamma pass rates from relevant studies on proton and photon applications, which were, therefore, not fully comparable to our application and highlighted the need for more studies on CIRT. The range shift analysis presented median RS within the clinical threshold (i.e., 5 mm), but with critical results for patients P27 and P31 (median (IQR), 5.69 (6.97) mm and 3.09 (2.60) mm, respectively), because of the presence of unmatched air pockets (Figure 5b). In proton plans, a maximum RS value of 5.6 mm (5.68%) was reported [25], whereas in our case, the inconsistencies of air cavities led to RS values of up to 15.37 mm (i.e., RRS = 9.09%, patient P27). This aspect may be critical for CIRT application, and needs to be analyzed on a wider population.

Similarly, the highest dose discrepancies were mostly found in correspondence of the different disposition of air pockets between sCT and planning CT (Figure S5 in Supplementary Material S7). The maximum error for patient P27 was due to an incomplete reproduction of the kidney (Figure S6), which caused an overdose for duodenum (+5.7% D_02_) with respect to the prescribed dose (Supplementary Material S6). The dose artefacts on recalculation for patient P21 reported regions of high dose differences (Figure S7), but this issue was not correlated with the quality of the sCT and could be easily overcome by acquiring wider volumes.

The main limitation of the study was the lack of a proper ground truth to validate the proposed approach, which could not be fully compensated due to the poor performance of multi-modal DIR in the abdominal site. As such, the use of computational phantoms [37,38] to ensure the correspondence between CT and MRI scans will be considered in the future as an effective approach to validate the proposed network, as anticipated in this work (Supplementary Material S4), and enlarge the training dataset. In addition, although the three channel implementation allowed good performance of the net in such a complex anatomical site with limited data, manual segmentation can be demanding, especially for not well-contrasted structures in VIBE acquisitions, such as bone, and is definitely not suited for clinical application. Further steps could be, therefore, to include the acquisition of specific MRI sequences (e.g., ultrashort echo time, UTE) to facilitate bone segmentation or avoid channel separation in an improved version of the net [26]. We also expect that our results could be improved by increasing the dataset; in future analyses, we intend to include different respiratory phases to (i) achieve higher accuracy and limit errors in the reproduction of tissues, (ii) eliminate separation in the three channels, and (iii) derive synthetic respiratory-correlated 4DCT [10,41]. Finally, although our results were mainly affected by air-filling effects, the absence of the thermoplastic mask on the sCT could also have an impact [32]; as such, a uniform and pre-defined outline to the sCT could be applied [32], although it would not be an optimal countermeasure.

Despite the above-mentioned limitations, this work showed that the three-channel cGAN can generate accurate sCTs of the abdominal site that can support treatment planning, evaluation, and adaptation in CIRT. To the authors’ best knowledge, this work is the first analysis applied to the abdomen for CIRT, and thus represents a starting point for future in-depth analyses of the feasibility of MRI-only workflows in CIRT.

## Figures and Tables

**Table 1:** CIRT plan details for the patients used in the test set.

Patient	N of Beams	Prescribed Dose [Gy(RBE)]	Fractions	Position	Tumor Location
P17	1	43	10	Prone	Pancreas
P20	1	38.4	8	Prone	Pancreas
P21	2	57.6	12	Supine	Pancreas
P27	1	38.4	8	Prone	Pancreas
P31	1	48	10	Prone	Pancreas

**Table 2:** Average results for CV, test and MRI-Only procedures, compared to the literature. Average (St. Dev.). * Soft tissue. ** Lungs. *** Vertebral bodies. **** Bidirectional network.

		MAE_Body [HU]	RMSE [HU]	SSIM	PSNR [dB]	NCC	MAE_Air [HU]	MAE_Bone [HU]	MAE_Soft [HU]
Our work	CV	56.52 (8.31)	97.24 (17.56)	0.651 (0.043)	27.73 (1.23)	0.857 (0.054)	46.19 (6.30)	90.76 (7.86)	54.79 (8.98)
	TEST	57.08 (2.79)	99.69 (4.90)	0.67 (0.06)	27.64 (0.68)	0.92 (0.02)	54.42 (11.48)	86.03 (10.76)	55.39 (3.41)
	MRI-ONLY	88.22 (9.88)	181.10 (11.84)	0.59 (0.08)	20.99 (1.49)	0.76 (0.10)	279.01 (142.46)	154.87 (22.90)	75.00 (8.12)
Literature	[20]	78.71 (18.46)	-	-	-	-	-	152.71 (30.14)	53.89 (10.7)
	[24]	62(13)	-	-	30.0 (1.8)	-	104(38) **	167 (22)	36 (8) *
	[25]	72.48 (18.16)	-	-	22.65 (3.63)	0.92 (0.04)	108.06 (49.45)	216.81 (63.0)	58.62 (30.61)
	[26]	55.56 (2.27)	106.43 (11.45)	-	-	0.87 (0.03)		-	-
	[27]	-	-	-	-	-	-	-	90 (29)
	[28]	-	-	-	-	-	-	110.09 (29.23) ***	-
	[21]	89.8 (18.7)	-	-	27.4 (1.6)	-	-	-	-
	[23]	60.42 (2.27)	-	-	-	0.88 (0.03)	-	-	-
	[22]	6.30 (0.56) ****	-	0.90 (0.42)	-	-	-	-	-

## Data Availability

Data are unavailable due to privacy and ethical restrictions.

## Supplementary Materials

The following supporting information can be downloaded at: https://www.mdpi.com/article/10.3390/bioengineering10020250/s1, Figure S1: Average MAE values on the three channels, for each batch tested; Figure S2: Coronal view of the XCAT phantom (A. original, B. with gaussian noise), ComBAT phantom (C) and the resulting synthetic CT (D); Figure S3: A. Histogram representing the HD 95th percentile. B. Histogram relative to the CoM distances. C. Histogram of Dice coefficient; Figure S4: DVH graphs comparing original plan (solid line) with sCT-based recalculations (dotted line); Figure S5: CT, sCT and dose difference maps for the test patients. The transversal slice shown contains the pixel with maximum error (black dot). Red arrows show the possible causes; Figure S6: Coronal view of P27, showing an error in the reproduction of the kidney, that affects the dose distribution; Figure S7: Dose recalculation on patient P21, coronal view. The discrepancy in the MRI-CT FoV causes dose artefacts in the recalculation; Figure S8: Representative range shift analysis on patient P17. The graph shows the Dose and HU profiles evaluated along the white line, as shown on CT and sCT below. The horizontal black line represents the 80% of the peak of dose; Table S1: DIR vs No-DIR average metrics results on the test set (St. Dev.); Table S2: Cross validation results, Table S3: Results of the network validation on the phantom; Table S4: Geometrical evaluation of kidney’s segmentations; Table S5: D_2%_ Relative errors on each OAR. The values are referred to the prescribed dose; Table S6: Dose difference median values [IQR] for the test patients on the whole plan; Table S7: TOST-P test of equivalence P-values; Table S8: Average Gamma pass rates (St. Dev.) of this work and literature references; Table S9: Median (IQR) RS and RRS evaluated over 10 transversal slices for each beam. Ap = Anterior-posterior. L = Lateral.
