# Supplementary material for: Synthetic CT in Carbon Ion Radiotherapy of the Abdominal Site
Source: Bioengineering (Basel). 2023 Feb 14;10(2):250. doi: 10.3390/bioengineering10020250 (PMC9951997; doi:10.3390/bioengineering10020250)
Supplement: Supplementary file 1 [file bioengineering-10-00250-s001.zip › bioengineering-2190090-supplementary.pdf]

bioengineering

Supplementary material

Synthetic CT in Carbon Ion Radiotherapy of the abdominal site

Giovanni Parrella [1,*], Alessandro Vai [2], Anestis Nakas [1], Noemi Garau [1], Giorgia Meschini [1], Francesca Camagni [1], Silvia Molinelli [2], Amelia Barcellini [3,4], Andrea Pella [5], Mario Ciocca [2], Viviana Vitolo [3], Ester Orlandi [6], Chiara Paganelli [1] and Guido Baroni [1]

1 Department of Electronics, Information and Bioengineering, Politecnico di Milano, Piazza Leonardo da Vinci 32, 20133 Milan, Italy
2 Medical Physics Unit, National Center of Oncological Hadrontherapy (CNAO), Strada Campeggi, 53, 27100 Pavia, Italy
3 Radiotherapy Unit, National Center of Oncological Hadrontherapy (CNAO), Strada Campeggi, 53, 27100 Pavia, Italy
4 Department of Internal Medicine and Medical Therapy, University of Pavia, 27100 Pavia, Italy
5 Bioengineering Unit, National Center of Oncological Hadrontherapy (CNAO), Strada Campeggi, 53, 27100 Pavia, Italy
6 Clinical Unit, National Center of Oncological Hadrontherapy (CNAO), Strada Campeggi, 53, 27100 Pavia, Italy
* Correspondence: giovanni.parrella@polimi.it; Tel.: +39-02-2399-18-9022

SUPPLEMENTARY MATERIALS

S1 - ASSESSING THE NEED OF DIR

One of the most critical aspects when training the network for sCT generation is the anatomical correspondence between CT and MRI slices, especially in terms of air pockets consistency and breathing phase (organ motion). Although the two scans were acquired within the same day, anatomical misalignments between corresponding air pockets and soft tissues may have occurred in two corresponding CT-MRI slices because of organ motion. For this reason, a common approach is to apply Deformable Image Registration between CT and MRI to compensate for any internal motion.

Since in our study, for some patients, relevant DIR inaccuracies were present, we investigated the impact of DIR on the ability of the cGAN in mapping CT numbers into MRI values. In this regard, two training datasets were built starting from the same 32 volumes, one characterized by the application of DIR ("DIR" dataset), the other without DIR ("No-DIR" dataset).

The net was therefore trained separately on "DIR" and "No-DIR" training datasets and tested on a common "No-DIR" test datasets. Similarity metrics between sCT and target CT were evaluated to assess which training procedure was more effective.

The results between DIR and No-DIR (**Table S1)** were very similar in terms of PSNR, NCC and SSIM. As such, the network trained with the "No-DIR" dataset was used for the following steps, since it consisted in a faster and more reproducible workflow.

Table S1 DIR vs No-DIR average metrics results on the test set (St. Dev.).

	MAE [HU]	RMSE [HU]	SSIM	PSNR [dB]	NCC	MAE_air [HU]	MAE_bone [HU]	MAE_soft [HU]
**DIR**	58.01<br>(3.04)	101.5<br>(7.63)	0.64<br>(0.05)	26.14<br>(0.58)	0.91<br>(0.03)	36.26<br>(7.61)	85.64<br>(10.07)	57.14.<br>(.98)
**No DIR**	57.08<br>(2.79)	99.69<br>(4.90)	0.67<br>(0.06)	27.64<br>(0.68)	0.91<br>(0.03)	54.42<br>(11.48)	86.03<br>(10.76)	55.39<br>(3.41)
**MRI-ONLY**	88.22<br>(9.88)	181.10<br>(11.84)	0.59<br>(0.08)	20.99<br>(1.49)	0.76<br>(0.10)	279.01<br>(142.46)	154.87<br>(22.90)	75.00<br>(8.12)

S2 - CROSS VALIDATION: HYPERPARAMETERS OPTIMIZATION

The six-folds cross validation was used to optimize the discriminator learning rate to avoid any overfitting issue with the training process, and to check which batch size best fitted for this specific purpose. The structure of the network was kept as in the reference work from Isola et al.

Table S2 Cross validation results

Batch	LR Disc	MAE [HU]	RMSE [HU]	SSIM	PSNR [dB]	NCC	MAE_air [HU]	MAE_bone [HU]	MAE_soft [HU]
**1**	**$2\ 10^{-06}$**	56.82<br>(8.18)	97.18<br>(18.24)	0.652<br>(0.047)	27.13<br>(1.09)	0.857<br>(0.055)	49.57<br>(7.47)	90.64<br>(7.50)	55.00<br>(8.78)
	**$2\ 10^{-07}$**	56.52<br>(8.31)	97.24<br>(17.56)	0.651<br>(0.043)	27.73<br>(1.23)	0.857<br>(0.054)	46.19<br>(6.30)	90.76<br>(7.86)	54.79<br>(8.98)
**2**	**$2\ 10^{-06}$**	56.41<br>(7.20)	96.72<br>(16.18)	0.659<br>(0.044)	27.77<br>(1.25)	0.855<br>(0.053)	50.83<br>(5.71)	91.92<br>(8.26)	54.52<br>(7.75)
	**$2\ 10^{-07}$**	56.19<br>(7.00)	97.34<br>(16.08)	0.651<br>(0.045)	27.82<br>(1.24)	0.854<br>(0.055)	50.79<br>(5.56)	90.65<br>(8.30)	54.34<br>(7.52)
**4**	**$2\ 10^{-06}$**	57.72<br>(7.04)	100.06<br>(16.30)	0.664<br>(0.050)	27.85<br>(1.30)	0.850<br>(0.049)	56.97<br>(7.71)	95.64<br>(7.63)	55.52<br>(7.53)
	**$2\ 10^{-07}$**	56.81<br>(6.43)	97.74<br>(13.56)	0.620<br>(0.042)	27.47<br>(1.04)	0.851<br>0.053)	49.66<br>(5.52)	93.18<br>(8.23)	54.89<br>(6.89)
**1**	**$2\ 10^{-04}$**	55.34<br>(6.37)	94.04<br>(13.48)	0.66<br>(0.05)	25.77<br>(0.82)	0.861<br>(0.05)	44.54<br>(6.93)	90.97<br>(6.68)	53.53<br>(6.86)

None of the parameters have shown a great impact on the network results. Nonetheless, the average MAEs on the three channels were slightly better in case of batch = 1 (**Figure S1**). These results were supported by the accurate optimization by Isola, which has also suggested the batch=1 to be the best option for Image-to-image translation purposes. Therefore, this parameter was kept equal to one in this work.

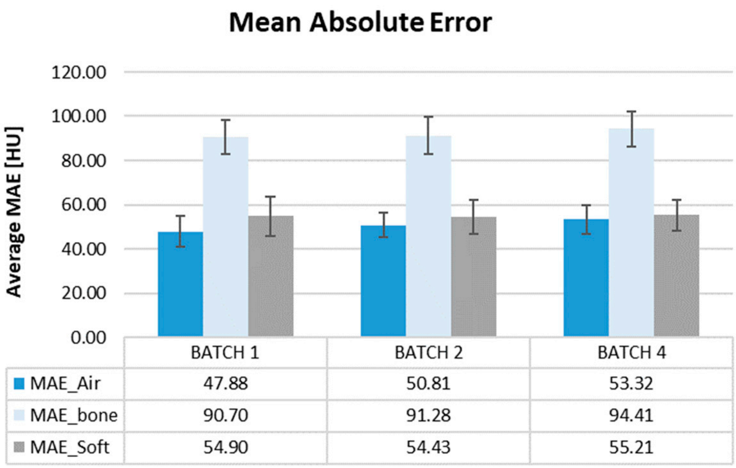

Figure S1 Average MAE values on the three channels, for each batch tested.

The learning rates proposed by Isola et al. (i.e. 2 10^{-4}) caused the discriminator loss function to frequently get stuck at ~0.6 after few epochs (i.e. 1 or 2 epochs), and the discriminator therefore stopped training following the adversarial paradigm. As such, a learning rate of 2*10^{-7} was set.

S3 - VOLUMES EVALUATIONS

Synthetic CT volumes from the test set were evaluated on the base of multiple similarity and geometrical metrics which measure the quality of the synthetic images from the pixel level to the whole image level.

In this work, all results have been evaluated on the base of reassembled volumes. Indeed, even the evaluation of MAE on the three channels was performed applying the corresponding air/bone/soft masks on the target CT and on the reassembled synthetic CT. The background was always excluded.

Similarity metrics

Mean Absolute Error – MAE

$$MAE = \frac{1}{N}\sum_i^N |x_i - y_i| \quad \text{with N pixels} \qquad (S1)$$

Root Mean Squared Error - RMSE

$$RMSE = \sqrt{MSE} = \sqrt{\frac{1}{N}\sum_i^N (x_i - y_i)^2} \quad \text{with N pixels} \qquad (S2)$$

Structural Similarity Index – SSIM

$$\text{SSIM (X,Y)} = \frac{(\mu_X\mu_Y + C_1)(\sigma_{XY} + C_2)}{(\mu_X^2 + \mu_Y^2 + C_1)(\sigma_X^2 + \sigma_Y^2 + C_2)} \qquad (S3)$$

Where X and Y are the two images, µX and µY are the two mean intensity values, σx2 and σy2 are the variances of the image X and Y, while σXY is the covariance.

Parameters C1 and C2 were defined to stabilize the index and they usually assume the values C1=0.0001 and C2=0.0009.

Normalized Cross Correlation – NCC

$$NCC = \frac{1}{N}\sum_{x,y,z}\frac{(CT(x,y,z)-\mu_{CT})(sCT(x,y,z)-\mu_{sCT})}{\sigma_{CT}\sigma_{sCT}} \quad \text{with N pixels} \tag{S4}$$

With CT(x,y,z) and sCT(x,y,z) being the CT and sCT values of the pixel at the coordinates (x,y,z), μ the average value and σ the standard deviation.

Peak Signal to Noise Ratio – PSNR

$$PSNR = 10\ \log_{10}\frac{MAX^2}{MSE} \tag{S5}$$

With MAX being the dynamic range of the pixels' value (2047 in this work). The results are expressed in Decibels [dB].

Geometrical metrics

Hausdorff Distance – HD

$$HD(X,Y) = \max\{sup_{x\in X}\ inf_{y\in Y}\ d(x,y)\,, sup_{y\in Y}\ inf_{x\in X}\ d(x,y)\,\} \tag{S6}$$

In this work, it was evaluated the 95th percentile of the Hausdorff Distance, to avoid outliers that would have biased the results.

Dice coefficient – DSC

$$DC = 2\frac{|X \cap Y|}{|X| + |Y|} \tag{S7}$$

Centre of mass distance - CoMD

The x coordinate of CoM for a binary image is calculated as:

$$CoM_x = \frac{\sum_i^N x_i}{N} \tag{S8}$$

Where xi is the coordinate of each of the N pixel having a value equal to 1. Similarly, the other coordinates are calculated on the Y and Z axes and the Euclidean distance is evaluated.

S4 – PHANTOM VALIDATION

An XCAT abdominal phantom with imaging parameters as those of patients data was used to generate the correspondent MRI volume through the ComBAT phantom. Then, the CT phantom was overlayed with gaussian noise to simulate a realistic ground truth CT. The resulting CT-MRI volumes pair (**Figure S2**) was then preprocessed as a normal input volume in this work, and used to validate the network on the basis of a matching ground truth, i.e. the net was tested giving as input the phantom MRI and comparing the sCT as output with the correspondent CT of the phantom. Results are

reported in **Table S3**. SSIM may be affected by the synthetic origin of such validation volumes, while NCC describes an acceptable reproduction of the structures.

Table S3 Results of the network validation on the phantom.

MAE_body [HU]	RMSE [HU]	SSIM	PSNR [dB]	NCC	MAE_air [HU]	MAE_bone [HU]	MAE_soft [HU]
73,32	130,82	0,55	24,97	0,89	77,93	167,36	66,11

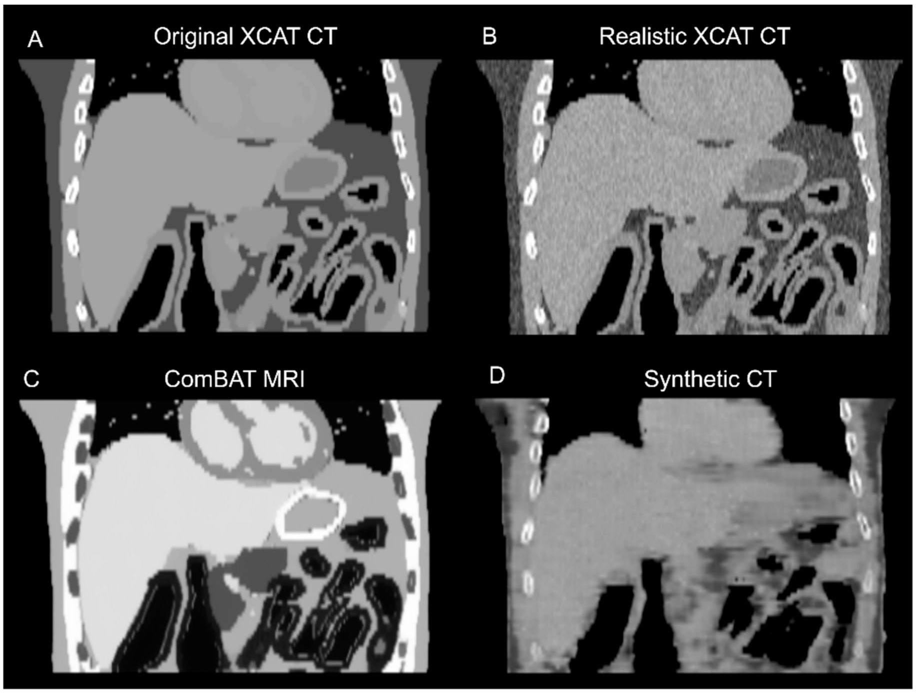

Figure S2 Coronal view of the XCAT phantom (A. original, B. with gaussian noise), ComBAT phantom (C) and the resulting synthetic CT (D)

S5 - GEOMETRICAL EVALUATION

Results of the geometrical evaluation on kidney's segmentations are presented in **Figure S3** and **Table S4**. From the results it was possible to classify the patients in two groups: one characterized by an evident mismatch between CT and MRI due to inter acquisition motion (patients P17, P21 and P20), the other characterized by a good overlap of structures between the two volumes. Therefore, in the latter case, only small differences were visible between sCT-MRI and sCT-CT evaluation, given the similarity of CT and MRI volumes.

Table S4 Geometrical evaluation of kidney's segmentations

	**Hausdorff distance 95th [mm]**			**Center of mass error [mm]**			**Dice Coefficient**		
	CT-MRI	sCT-CT	sCT-MRI	CT-MRI	sCT-CT	sCT-MRI	CT-MRI	sCT-CT	sCT-MRI
17	10.96	16.96	7.31	16.51	31.17	14.65	0.62	0.57	0.78
20	8.95	7.84	5.66	4.40	6.49	2.09	0.81	0.81	0.88
21	8.96	12.20	7.45	13.14	18.37	5.23	0.68	0.50	0.76
27	2.38	6.56	6.58	2.00	8.52	6.52	0.94	0.86	0.88
31	4.85	6.41	7.04	2.72	1.96	0.76	0.87	0.82	0.84
Mean	7.22	9.99	6.81	7.75	13.30	5.85	0.78	0.71	0.83
St.Dev	3.13	4.06	0.65	5.92	10.42	4.87	0.12	0.15	0.05

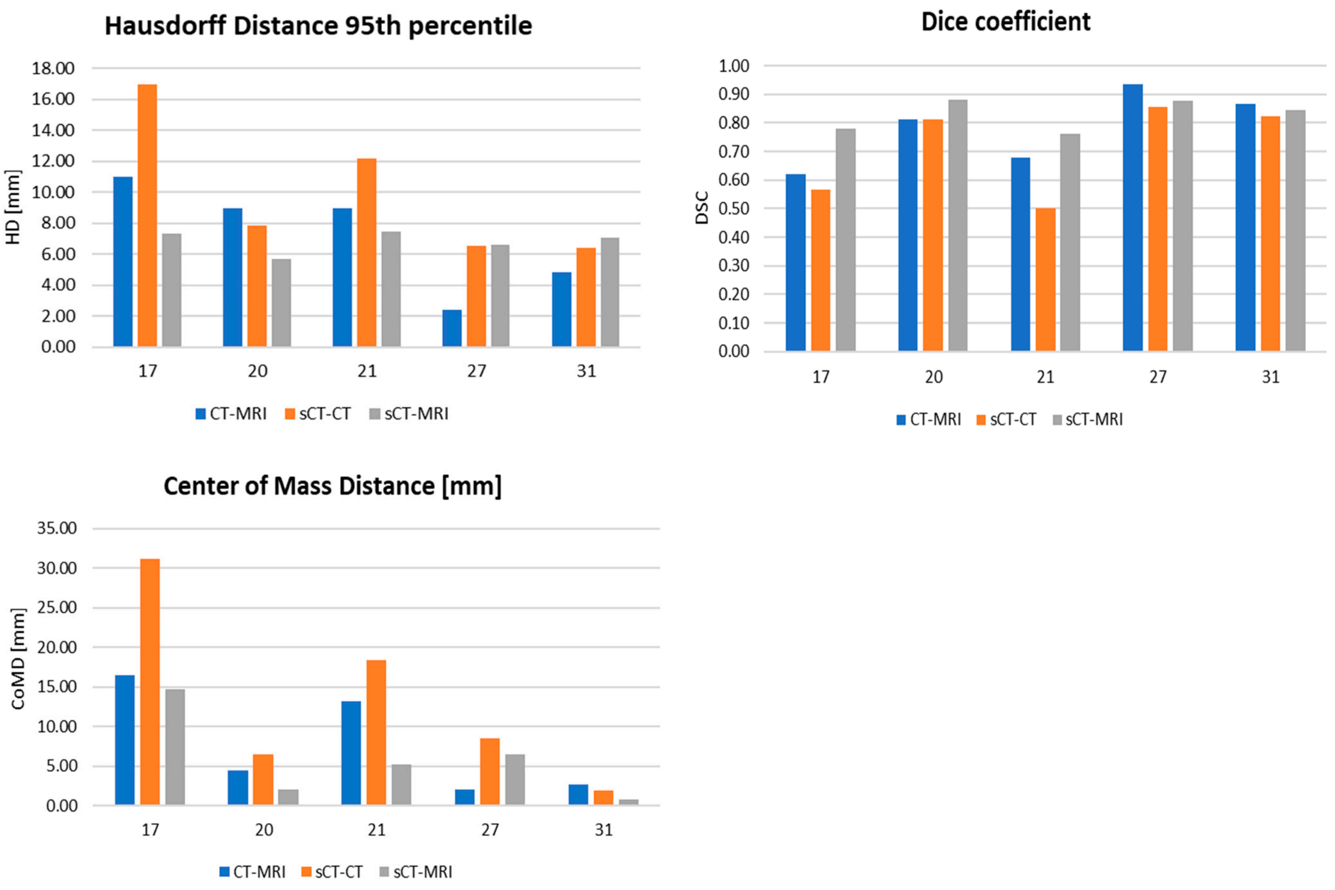

Figure S3 A. Histogram representing the HD 95th percentile. **B**. Histogram relative to the CoM distances. **C**. Histogram of Dice coefficient.

S6 - DVH

Figure S4 shown all the DVH from the test set. **Table S5** $D_{2\%}$ Relative errors on each OAR.

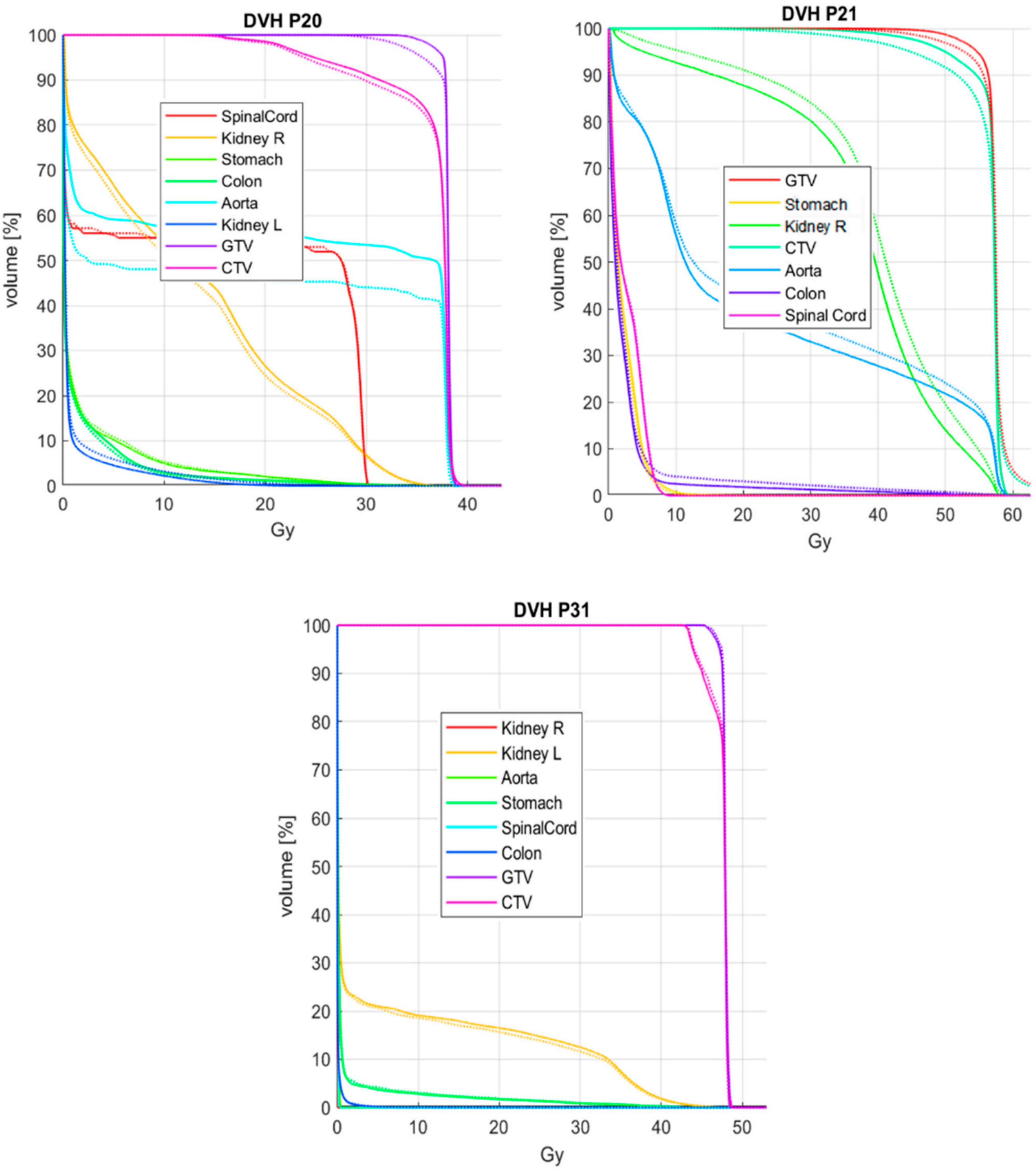

Figure S4 DVH graphs comparing original plan (solid line) with sCT-based recalculations (dotted line).

Table S5 $D_{2\%}$ Relative errors on each OAR. The values are referred to the prescribed dose.

Relative Error [%]		Kidney R	Kidney L	Aorta	Stomach	Colon	Spinal Cord	Duodenum
	P17	-2.51	0.02	-0.02	0.35	0.56	0.21	-9.93
	P20	-0.44	7.08	-0.68	0.96	-3.49	-0.03	-4.48
	P21	0.49	0.00	0.09	-1.20	26.35	0.16	-
	P27	-2.68	-0.78	-0.26	0.00	37.03	-0.16	5.7
	P31	0.00	-0.71	-0.06	4.15	0.04	-0.02	16.25
	**E_{mean}[%]**	-1.03(1.31)	1.12(0.58)	-0.19(0.27)	0.85(1.79)	12.10(16.41)	0.03(0.13)	1.89(10.01)

S7 – DOSE DIFFERENCE

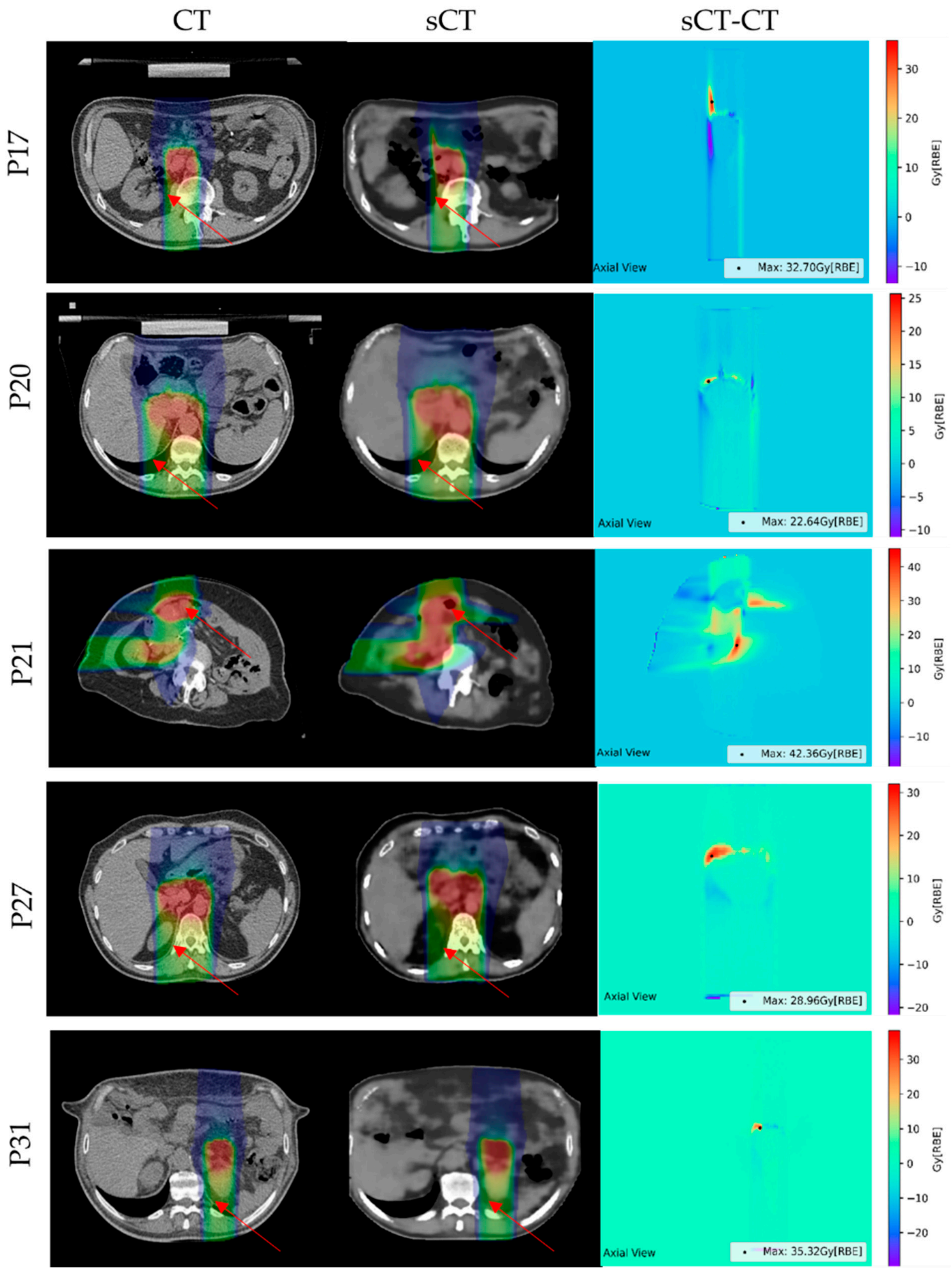

Figure S5 CT, sCT and dose difference maps for the test patients. The transversal slice shown contains the pixel with maximum error (black dot). Red arrows show the possible causes.

Figure S5 shows the dose difference map for the test patients, highlighting the maximum error (black dot), while **Table S6** contains the median values with the interquartile range and the maximum error, for each patient inside the body outline. **Figure S6** shows the error in the reproduction of the kidney, that affects the dose distribution in patient P27. In all the other cases, the maximum error was induced by inter acquisition motion of air pockets (**Figure S5**). To be noted, the limited field of view of MRI introduced dose artefacts on recalculation for patient P21 (**Figure S7**), but this can be overcome by acquiring wider volumes.

Table S6 Dose difference median values [IQR] for the test patients on the whole plan

	P17	P20	P21	P27	P31
Dose Difference Median [IQR] Gy[RBE]	0.0044 [-0.0042,0.0368]	0.0002 [-0.0148,0.0234]	-0.0055 [-0.0868,0.0711]	-0.0009 [-0.0110,0.0070]	0.0021 [-0.0151,0.0457]
Dose Difference Max Error Gy[RBE]	32.71	22.64	42.36	28.97	35.33

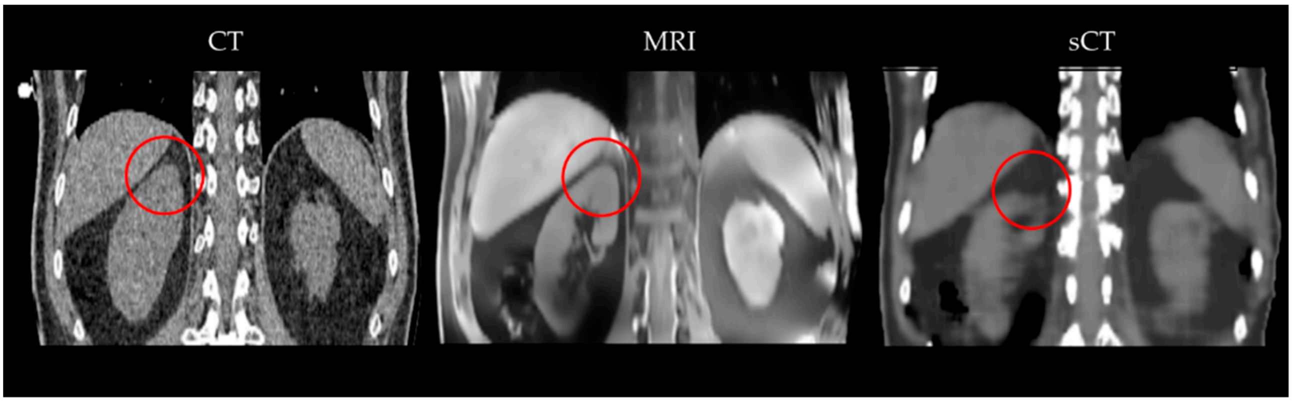

Figure S6 Coronal view of P27, showing an error in the reproduction of the kidney, that affects the dose distribution.

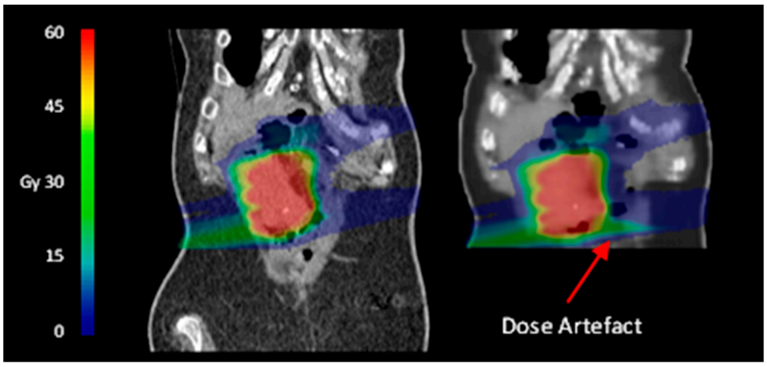

Figure S7 Dose recalculation on patient P21, coronal view. The discrepancy in the MRI-CT FoV causes dose artefacts in the recalculation

S8 - TOST-P STATISTICS

A two one-sided test of equivalence for paired samples (TOST-P) was used to compare the DVH results, considering a confidence interval of 95% and an equivalence interval of ±0.5%.

The p-values from the TOST-P test of equivalence are shown in **Table S7** for each district.

Table S7 TOST-P test of equivalence P-values

GTV	CTV	Kidney_R	Kidney_L	Aorta	Stomach	Colon	Spinal Cord	Duodenum
9.51e-07	8.88e-05	9.70e-08	1.70e-07	1.48e-05	2.01e-06	5.37e-3	4.41e-06	1.82e-3

S9 - GAMMA ANALYSIS

Table S8 shows the average global gamma pass rates of this work with respect to some references in literature, taking into account that the works by Y.Liu and Florkow are the only available studies relative to particle therapy plans (i.e. protons) and therefore are more relevant for our purposes.

Table S8 Average Gamma pass rates (St. Dev.) of this work and literature references

Reference	Plan	γ1%,1 mm [%]			γ2%,2 mm [%]			γ3%,3 mm [%]		
		D>10%	D>50%	D>90%	D>10%	D>50%	D>90%	D>10%	D>50%	D>90%
This work	Carbon	65.40 (13.3)	69.77 (14.1)	72.05 (11.5)	87.21 (12.1	88.23 (11.1	89.67 (8.9)	93.94 (6.7)	93.96 (6.2)	94.88 (4.9)
Y. Liu,2019	Proton	90.76 (5.94)	-	-	96.98 (2.93)	-	-	99.37 (0.99)	-	-
Florkow,2020	Proton	-	-	-	96.2 (4.0)	98.6 (2.0)	99.2 (1.1)	-	-	-
Cusumano, 2020	Photon	90.8 (4.5)	-	-	98.7 (1.1)	-	-	99.8 (0.2)	-	-
Olberg, 2021	Photon	-	-	-	-	-	-	93.9 (9.8)	-	-

S10 – RANGE SHIFT ANALYSIS

Table S9 shows the Median (IQR) RS and RRS evaluated over 10 transversal slices for each beam**. Figure S8** shows the dose and HU profile along the line, for patient P17.

Table S9 Median (IQR) RS and RRS evaluated over 10 transversal slices for each beam. Ap = Anterior-posterior. L = Lateral.

	P17	P20	P21-ap	P21-l	P27	P31
**RS** [mm]	0.78(1.10)	0.44(1.99)	-0.32(0.32)	-2.12(4.69)	5.69(6.97)	3.09(2.60)
**RRS** [%]	0.49(0.71)	0.25(1.07)	-0.27(0.27)	-1.29(2.9)	3.39(3.97)	2.03(1.86)

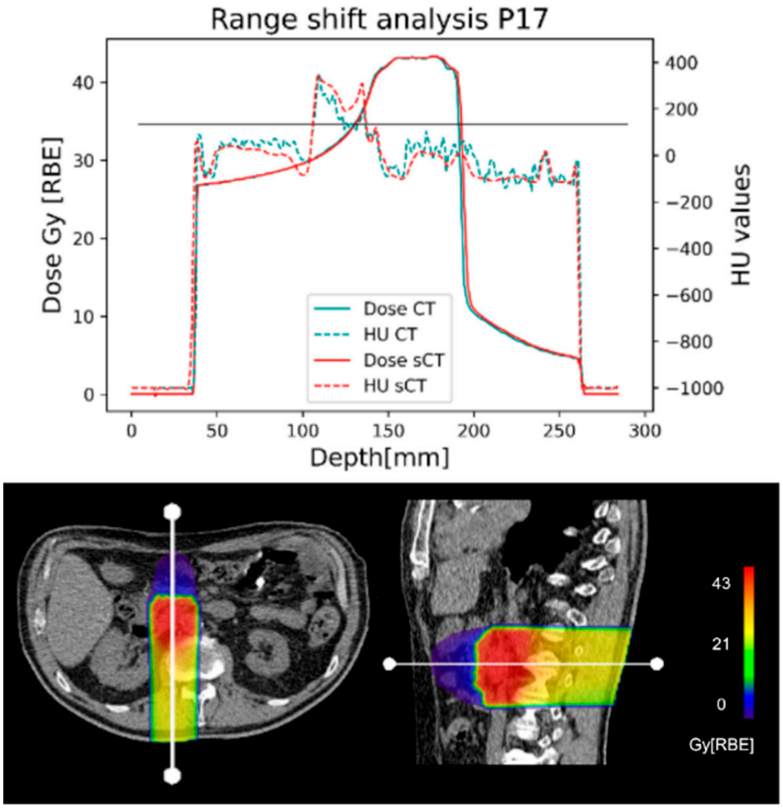

Figure S8 Representative range shift analysis on patient P17. The graph shows the Dose and HU profiles evaluated along the white line, as shown on CT and sCT below. The horizontal black line represents the 80% of the peak of dose.
